# Enhancing Resolution for Flash LiDAR with Multi-View Imaging Optics and Range Image Tiling

**DOI:** 10.3390/s25113288

**Published:** 2025-05-23

**Authors:** Jui-Hsiang Yen, Shao-Jung Li, Zih-Ying Fang, Cheng-Huan Chen

**Affiliations:** 1 Department of Photonics, College of Electrical and Computer Engineering, National Yang Ming Chiao Tung University, 1001 University Road, Hsinchu 300, Taiwan; rick.c@nycu.edu.tw (J.-H.Y.); monkey310276.c@nycu.edu.tw (S.-J.L.); judy3761.ee12@nycu.edu.tw (Z.-Y.F.); 2 IGIANT Optics Company, No. 13-1, Zhonghua Road, Hukou Township, Hsinchu 303, Taiwan

**Keywords:** flash LiDAR, time of flight, range image tiling, depth sensing calibration

## Abstract

Flash LiDAR can be used to capture the depth information or contour profile of a target area in one shot and, therefore, has become an important feature in depth sensing devices. Current flash LiDAR devices still suffer from resolution issues due to difficulties in reducing the pixel size of the array sensor. In this paper, a solution has been proposed to resolve this resolution issue by introducing multi-view imaging optics into the flash LiDAR device. Together with scanning illumination over the target area and range image tiling, a fourfold increase in spatial resolution has been achieved, and the working concept can be further extended for resolution enhancement via array expansion. A calibration procedure and shielding mechanism have also been developed to compensate for the issues of crosstalk and stray light, ensuring the quality of the tiled range image. The prototype and corresponding experiment have demonstrated the feasibility of the proposed architecture as an optical solution for enhancing the resolution of flash LiDAR.

## 1. Introduction

The use of Light Detection and Ranging (LiDAR) has been growing quickly in recent years due to the demand for compact and low-cost solutions with applications in auto-vehicles [1,2], smart city surveillance [3], and industrial automation [4,5]. The majority of early-stage LiDAR devices use a mechanically rotating mirror to scan a laser beam and acquire high-resolution 3D point cloud data on the target area. These systems are bulky, costly, and consume quite a large amount of power. Recent developments have moved toward the design of miniature solid-state LiDAR devices, such as MEMS-based LiDAR, flash LiDAR, optical phase array (OPA)-based LiDAR [6,7,8,9,10], etc. MEMS- and OPA-based LiDAR systems use micro-mirrors and optical phase arrays, respectively, to manipulate or make the scan of the laser beam. Although there is no mechanical movement, a scanning mechanism is still required, and the frame rate for a scene with a large field of view can be slow. Therefore, flash LiDAR, which can acquire depth information of a target area in one shot, has been attracting attention. The overall structure is similar to a traditional optical imaging system but with an additional illumination light source synchronized with the sensing array. There is no mechanical movement involved, not even a scanning mechanism for the laser beam. This feature endows flash LiDAR with the potential to be made with high reliability and low cost, and a miniature form can be created for embedded devices, making it especially suitable for application in mobile systems [11]. Nevertheless, the resolution of flash LiDAR is still largely limited by the available technologies for reducing pixel size and raising signal processing speed [12].

**Figure 1:**
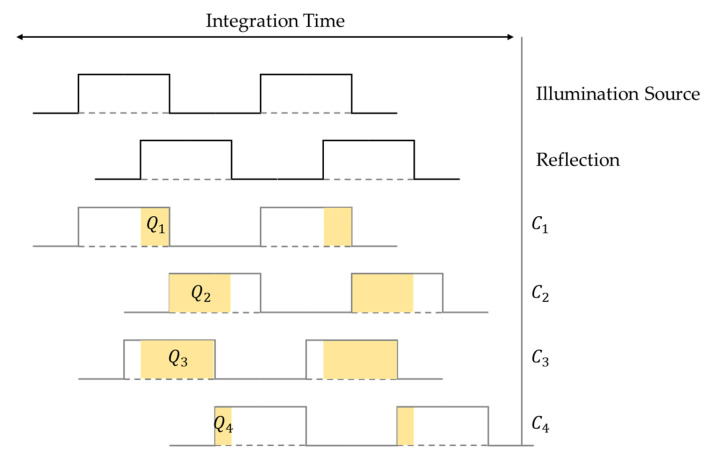
Timing diagram of the indirect time-of-flight concept.

Flash LiDAR uses the concept of time of flight (ToF) to acquire the 3D depth information of a target area. TOF devices measure the time it takes for light to travel from the emitter to the target and then back to the sensor, so as to evaluate the corresponding distance. There are two fundamental elements of ToF, namely the direct and indirect ToF. Direct ToF uses a pulsed illumination source and measures the traveling time of the pulse directly. Indirect ToF uses a continuous-wave (CW) laser source and measures the phase between several samples in a time sequence. There are four samples in total and the phase difference between two samples is 90 degrees. Figure 1 shows the timing diagram and the electric charge (*Q*_1_, *Q*_2_, *Q*_3_, and *Q*_4_) accumulated on four pixel taps (*C*_1_, *C*_2_, *C*_3_, and *C*_4_). With these data, the phase shift between the emitting and detecting event φ and the distance *d* can be evaluated with Equations (1) and (2), respectively [13].

(1) $$\varphi =\arctan(\frac{Q_{3}-Q_{4}}{Q_{1}-Q_{2}})$$

(2) $$d=\frac{c}{4\pi f}\varphi$$

where *f* is the modulation frequency, and *c* is the speed of light.

**Figure 2:**
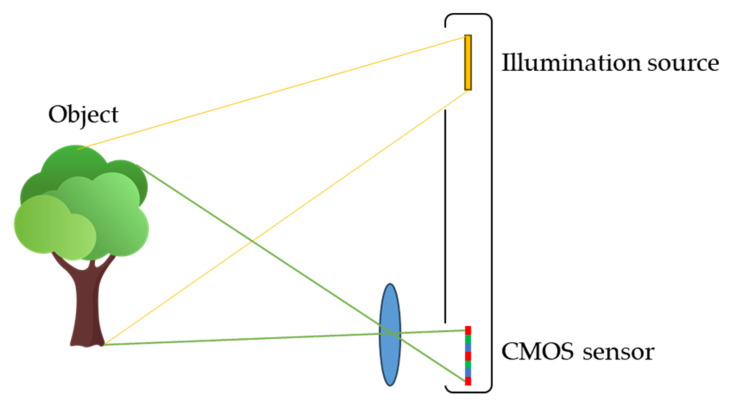
Schematic light path of the flash LiDAR device.

Figure 2 shows a schematic diagram of the light path for a flash LiDAR detecting system. The light path from the object to the sensor is simply an imaging relationship, and therefore, the spatial resolution of the system is determined by the resolution of the sensor. The major resolution specification in the market is quarter VGA (QVGA; 320 × 240) and the latest development can reach Video Graphics Array (VGA; 640 × 480) standard only.

Comparing all different kinds of ToF devices, the resolution of flash LiDAR is considered relatively low. Major improvements in this area are focused on sensor hardware and post-signal processing, while keeping the same imaging optics [6,14,15,16,17,18,19,20]. In this paper, an alternative solution for enhancing the resolution of flash LiDAR systems has been proposed, and its focus is on maintaining the imaging optics while ensuring the use of currently available flash LiDAR sensors.

## 2. System Architecture and Working Concept

**Figure 3:**
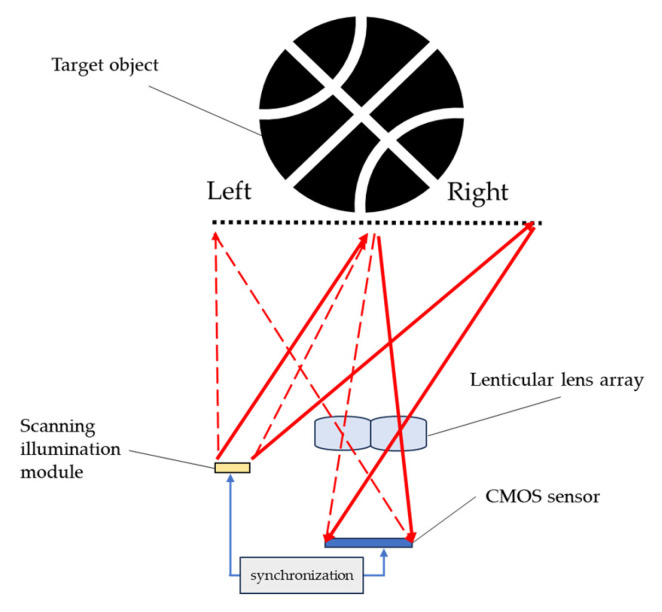
Multi-view imaging optics with synchronized scanning illumination [21].

The imaging optics in Figure 2 directly form the image of the object and fill the sensor. The whole-object image uses the full resolution of the sensor. However, if the field of view of the imaging optics is reduced by half and only half of the object can be captured in one shot, then only half of the object image uses the full resolution of the sensor. If two shots are taken for the object—one for the left part and the other for the right part—and then those two shots are tiled together to make the full image of the object, the whole-object image is then equivalently captured with double the resolution of the sensor. Figure 3 shows the schematic diagram of the system architecture utilizing the above-mentioned concept without the need for rotating the imaging optics to capture different parts of the object image. A lenticular lens array can form the image of different parts of the target object onto the same sensor, as shown with the red light path in Figure 3. In this case, the target object is divided into two parts: the right and left part. Which part of the object is captured depends on which part of the object is illuminated. Therefore, the illumination pattern of the scanning illumination module scans between the right and left parts of the target object and is synchronized with a complementary metal-oxide semiconductor (CMOS) sensor to determine which part of the object is being illuminated. The synchronization between the illumination and the sensing modules is enabled by a built-in mechanism in the ToF device for evaluating depth or distance information. The additional synchronization between the illumination scanning and the sensor is then used as the reference for post-image tiling.

## 3. Prototype Design and Establishment

**Figure 4:**
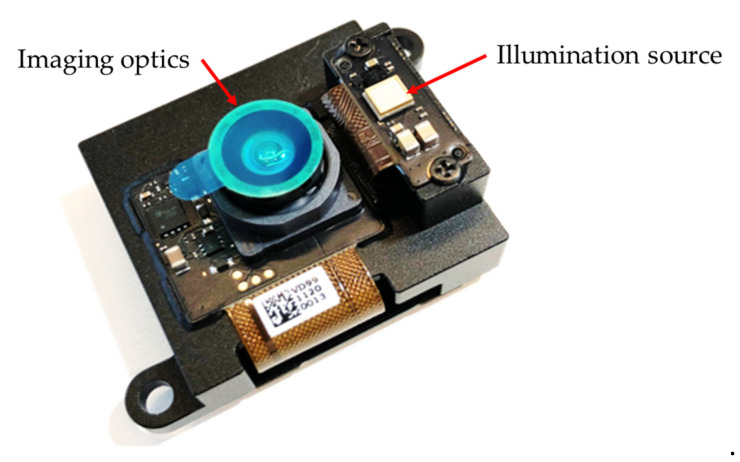
AD-FXTOF1-EBZ flash LiDAR device.

The flash LiDAR system used for the prototype is AD-FXTOF1-EBZ, provided by Analog Devices, Inc. (Wilmington, MA, USA), as shown in Figure 4, with the illumination source and the imaging optics highlighted. The wavelength of the illumination is 940 nm. The major specifications of the flash LiDAR device are listed in Table 1.

With this commercial flash LiDAR device, the remaining parts needed to establish the system shown in Figure 3 are the illumination scanning optics and the lens array imaging optics.

### 3.1. Illumination Scanning Optics

**Figure 5:**
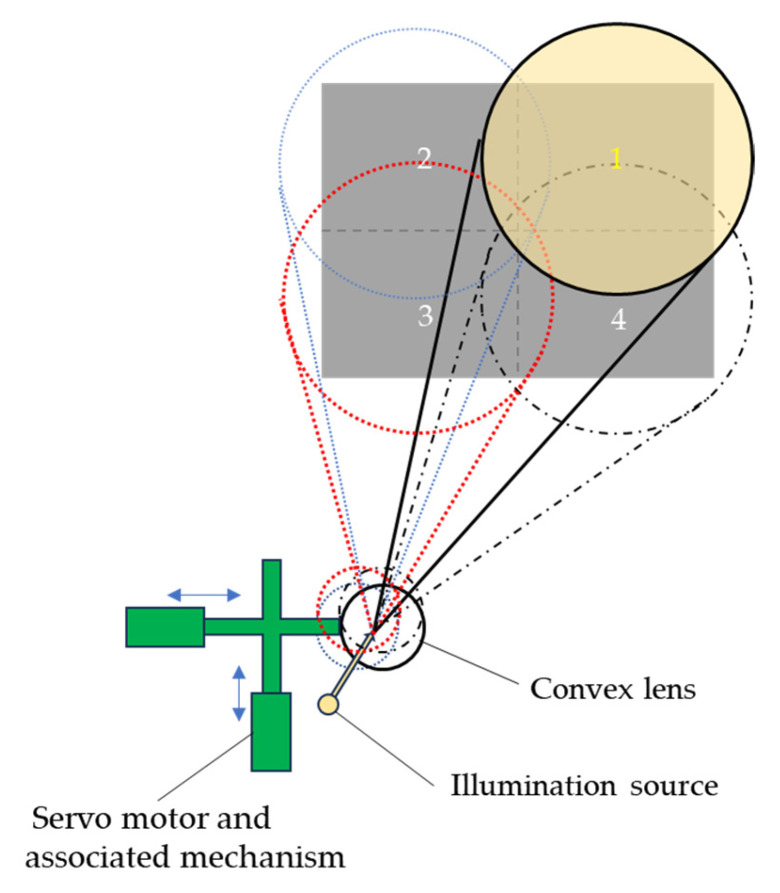
Illumination scanning optics. The four numbered circles (1–4) represent the sequential quadrants of the target area scanned by the illumination source. The arrows indicate the horizontal and vertical translation directions of the lens module driven by the servo motor.

As the illumination has to be synchronized with the sensor to function as a LiDAR system, a built-in illumination source has to be used. The approach taken to make a scan of this built-in illumination source was to put a convex lens in front of the exit of the illumination source and make a scan of the illumination by shifting the lens element with a servo motor and an associated mechanism. Figure 5 shows a schematic diagram to illustrate the sequential scanning of the illumination over the four quadrants of the target area [21].

### 3.2. Multi-View Imaging Optics

**Figure 6:**
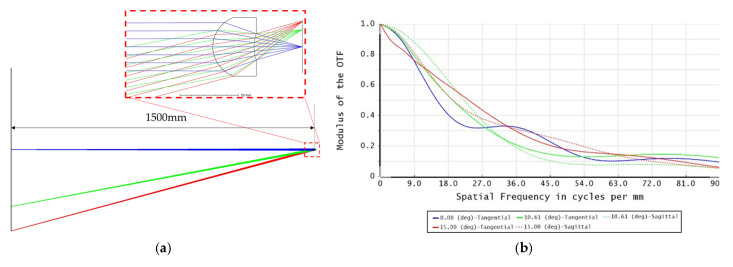
Simulation of the lens element: (**a**) simulation light path of the lens element; (**b**) MTF of the lens elements.

To make a multi-view imaging system, the original imaging optics of the flash LiDAR device should be replaced with a lens array. In this case, the target detecting area was divided into four quadrants, i.e., the imaging optics involved a 2 × 2 lens array. The field of view of each lens element covered 30 degrees on the diagonal for a target area 1500 mm away from the flash LiDAR device, as 1500 mm is within the medium range mentioned in the specification of the LiDAR. The target object size, i.e., the size of the single quadrant of the target area, was therefore 401.924 mm. The major specifications of each lens element are listed in Table 2. Figure 6a,b show the simulation light path and modulation transfer function (MTF) of the lens element from the ZEMAX^TM^ commercial ray tracing program.

**Figure 7:**
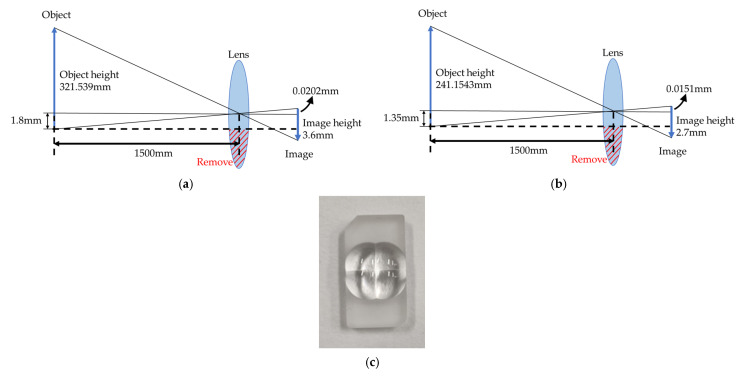
Schematic diagram of lens array: (**a**) lateral shift of lens in horizontal direction; (**b**) lateral shift of lens in vertical direction; (**c**) 2 × 2 lens array.

In order to make the imaging light path shown in Figure 3, each lens should be laterally shifted by the correct amount so as to make each quadrant of the target area just fill the whole active area of the sensor. Based on the specifications in Table 2 and ray tracing data, the shifts are 0.0202 mm and 0.0151 mm in the horizontal and vertical directions, respectively, as shown in Figure 7a,b. Figure 7c shows the prototype of the 2 × 2 lens array fabricated with precision diamond turning.

### 3.3. Flash LiDAR Device with Multi-View Imaging Optics

**Figure 8:**
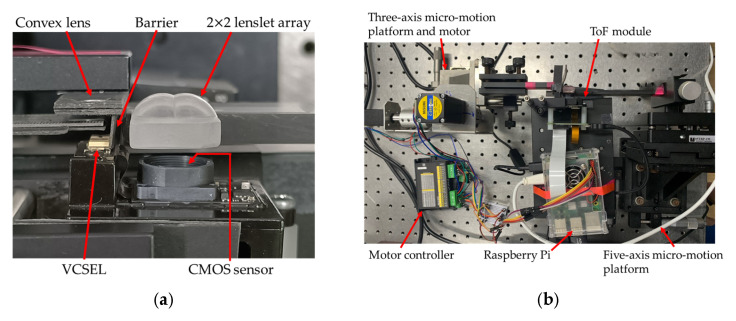
Prototype of the flash LiDAR system: (**a**) flash LiDAR device with multi-view imaging optics and illumination scanning optics; (**b**) system prototype with mechanics and electronic controller.

Figure 8a shows the prototype of the flash LiDAR device with the 2 × 2 lens array as the multi-view imaging optics in front of the CMOS sensor and a scanning convex lens as the scanning optics for the illumination source. Figure 8b shows all the system hardware including a five-axis precision mechanism for positioning the 2 × 2 lens array, a three-axis mechanism and associated motor controller for moving the convex lens to make the illumination scan over four quadrants of the target area, and a Raspberry Pi as the interface between the sensor and the computer.

## 4. Experiment and Calibration

**Figure 9:**
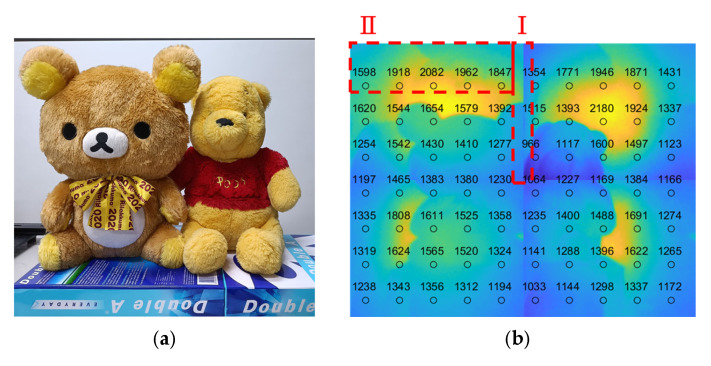
Target object and range image: (**a**) target object; (**b**) tiled range image of target object. Symbols I and II indicate the regions where discontinuities and incorrect depth evaluations were observed, respectively.

Applying the prototype shown in Figure 8, the object shown in Figure 9a, positioned 1500 mm away from the LiDAR sensor, was used as the target object for the experiment. An additional white Lambertian plate, 2300 mm away from the LiDAR, was used as the background. For the purpose of verifying the feasibility only, the illumination scanning optics were not synchronized with the CMOS sensor; four quadrant images were acquired one by one with manual control, and tiling of the range image was conducted off-line. The resultant tiled range image is shown in Figure 9b, where the numbers indicate the depth/distance information and different colors represent different depths/distances.

Several issues can be seen from Figure 9b. The first is that the depth is not smoothly varied across the tiling boundary of two adjacent range images, as shown by the area marked as I in Figure 9b. The second is the incorrect depth evaluation, as shown by the area marked as II in Figure 9b, where the depth should be constant. As for the slight discontinuity of the image at the tiling boundary, the reason is that there is a redundant area at the outmost active area of the CMOS sensor. This can be easily resolved by modifying the magnification ratio of the lens and is not a concern when evaluating this tiling technology. As the working concept of flash LiDAR is based on indirect ToF, as described in Section 1, the electric charge accumulation during a certain period will affect the evaluation of depth information. Therefore, the illumination will certainly influence or disturb the precision of depth evaluation. The sources of disturbing illumination are stray light and crosstalk, which can be resolved with proper shielding and calibration [22,23].

### 4.1. Stray Light Simulation and Elimination

**Figure 10:**
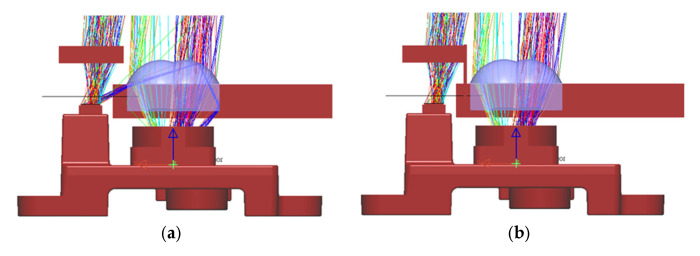
Simulation of light path: (**a**) original light path; (**b**) light path after adding light shield.

The ray tracing program LightTools was used for simulating the light path of the illumination source, and Figure 10a shows the original light path emitting from the illumination source and returning back into the CMOS sensor. It shows that there is stray light from the source directly propagating into the CMOS sensor through the total internal reflection on the surface of the lens array, mainly because there is no housing of the lens array as the original imaging optical module and the lens array are a little further away from the CMOS sensor. As a result, the depth measurement is severely affected, with most values being measured as zero due to the overwhelming influence of stray light. The approach to resolve this issue is to put a barrier in between the illumination source module and the imaging optical module, as shown in Figure 10b. The picture of the prototype shown in Figure 8a already has the light shield inserted, and the tiled range image shown in Figure 9b was also obtained with the light shield.

### 4.2. Crosstalk Evaluation and Simulation

**Figure 11:**
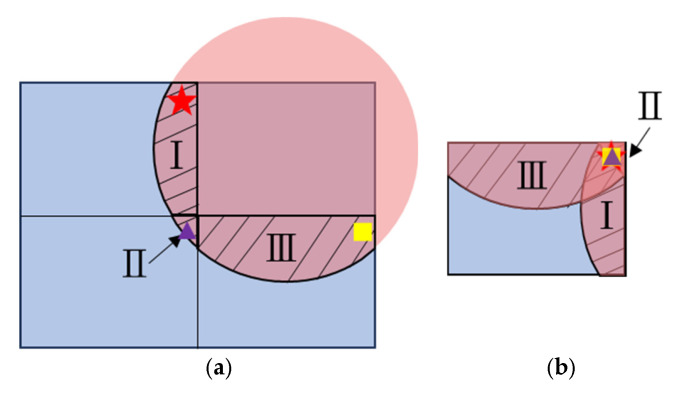
Crosstalk pattern with lens array imaging optics: (**a**) illumination area on target plane; (**b**) crosstalk on CMOS sensor. The red star, purple triangle, and yellow square represent the object points in regions I, II, and III respectively. Regions I, II, and III denote three distinct quadrants of the target area that partially overlap on the CMOS sensor.

As shown in Figure 5, the illumination pattern does not match well with the shape and size of each quadrant of the target area. This phenomenon is illustrated in Figure 11a, where the parts with over-illumination on the other quadrants are marked with I, II, and III. The red star, purple triangle, and yellow square represent the object points in area I, II, and III, respectively, which will be imaged onto the same pixel of the CMOS sensor with the 2 × 2 lens array, as illustrated in Figure 11b, where the same symbols (a red star, purple triangle, and yellow square) are used to indicate the corresponding image points on the CMOS sensor. In these overlapping regions on the CMOS sensor, each pixel of the sensor receives signals from multiple object points simultaneously. In particular, in the upper right corner of the sensor area, the pixels are exposed to signals from all four quadrants, leading to interference from four different depth values. This severe crosstalk effect distorts the depth evaluation, hence reducing the measurement accuracy and reliability.

**Figure 12:**
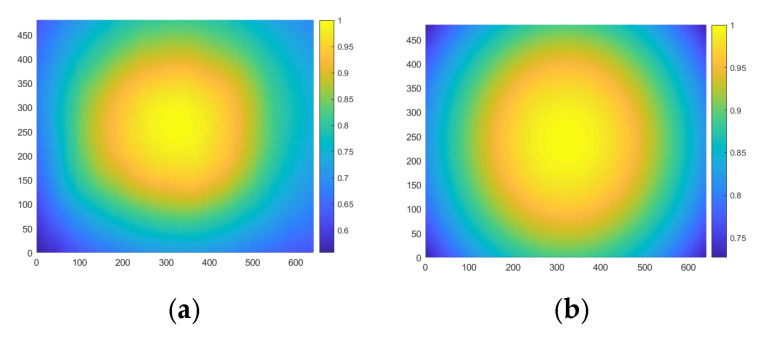
Intensity distribution of the illumination source: (**a**) measured intensity distribution of illumination pattern; (**b**) Gaussian distribution model of illumination pattern.

**Figure 13:**
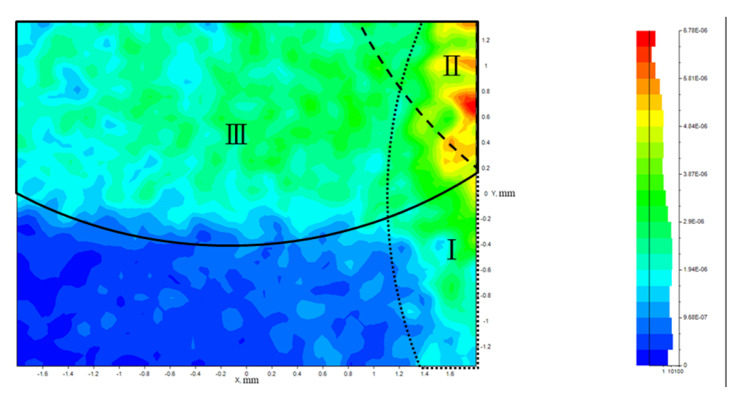
Simulation of crosstalk effect on the CMOS sensor for the first quadrant of the target area. Regions I, II, and III indicate areas affected by over-illumination on other quadrants, which is attributed to the crosstalk effect.

In order to build a model for quantitatively correcting the crosstalk, the intensity distribution of the illumination source was investigated. Figure 12a shows the measured intensity distribution of the illumination pattern, and Figure 12b is a Gaussian distribution model of intensity distribution. Due to the similarity between these two patterns, the Gaussian distribution has been taken as the quantitative model for illumination. Figure 13 shows the simulation of light distribution on the CMOS sensor with Gaussian illumination on the first quadrant of the target area, assuming the target is simply a flat reflective Lambertian surface. The regions marked as I, II, and III indicate areas affected by over-illumination on other quadrants, which is attributed to the crosstalk effect.

### 4.3. Calibration for Depth Evaluation

Because the original imaging optics of the flash LiDAR were replaced with a 2 × 2 lens array, all the optical parameters became different and the calibration for depth evaluation had to be rebuilt in response. Measured depth calibration is required for correlating the real depth with a big database, i.e., a look-up table (LUT) [18], based on measured data. Due to the use of a 2 × 2 lens array, the calibration actually involves the influence of crosstalk.

**Figure 14:**
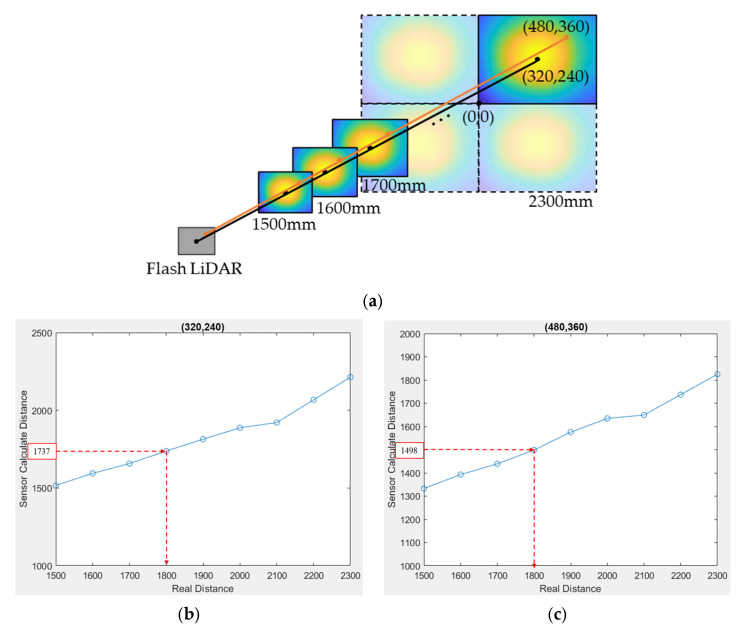
LUT database for depth calibration: (**a**) schematic diagram of setup for depth calibration; (**b**) LUT of the pixel (320,240) for first quadrant; (**c**) LUT of the pixel (480,360) for first quadrant.

The procedure to build up this LUT is to put a flat reflective Lambertian surface in front of the flash LiDAR system, at a distance from 1500 mm to 2300 mm away, which is the medium range of the flash LiDAR module. The step of movement is arbitrary, but a smaller step gives a higher precision calibration. Figure 14a shows a step of 100 mm for this procedure. At each distance, each pixel has its distance measured, and this measured distance should correspond to the real distance where the flat surface locates for that pixel. By taking this measurement at each step of distance, a look-up table correlating measured distance and real distance can be built up for each pixel. For the case of the 640 × 480-resolution CMOS sensor where the calibration was performed for all four quadrants at each distance step, there were 1,228,800 LUTs in total. Figure 14b,c show only the LUTs of the pixels numbered (320,240) and (480,360) for the first quadrant.

With the LUTs shown in Figure 14, $D_{Sensor\_measured}(M,N)$ in Equation (3) is taken as the measured depth, and the final calibrated depth $D_{corrected}\left(M,N\right)$ is obtained using the LUTs as follows:

(3) $$D_{corrected}\left(M,N\right)=LUT(D_{Sensor\_measured}(M,N))$$

**Figure 15:**
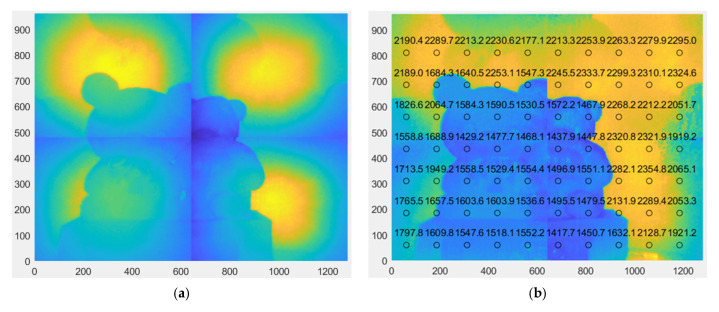
Tiled range image before and after calibration: (**a**) without LUT calibration; (**b**) with LUT calibration.

Figure 15a,b show the tiled range images without and with LUT calibration. They indicate significant improvement in the tiled image, especially at the boundary of the two adjacent quadrants. However, depth anomalies still exist at the outermost boundary regions, where the depth should be a constant of 2300 mm, due to crosstalk from multiple object points with different depths, as described in Section 4.2.

## 5. Discussion

**Figure 16:**
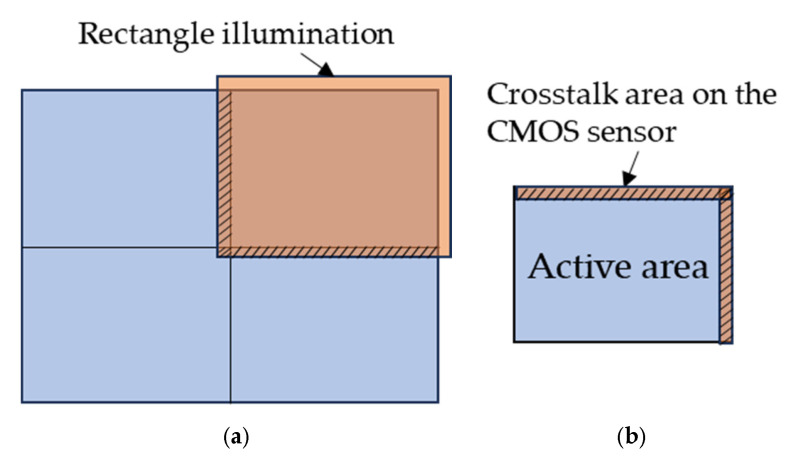
Reshaped illumination pattern for resolving crosstalk effect: (**a**) rectangular illumination on first quadrant of the target area; (**b**) crosstalk area on the CMOS sensor.

The depth anomaly in Figure 15b is especially severe for where the multiple object points with a large depth gap come to disturb the depth evaluation. As the disturbance resulting from crosstalk is highly dependent on the surface contour of the target object and surrounding environment, it is fully random and cannot be quantitatively analyzed and compensated for. Therefore, a solution that is able to fully eliminate crosstalk is essential. To address this issue, the illumination pattern has to be reshaped to match with the shape and size of the target area, in this case, a rectangular pattern to just fill one quadrant of the target object. As the illumination sources are normally coherent light for ToF devices, a well-designed diffractive optical element can easily reshape the laser beam to meet these diversified requirements. Even with a little over-illumination, as shown in Figure 16a, the crosstalk area on the sensor active area can be just a thin stripe boundary, as shown in Figure 16b. With this situation, the imaging optics can be designed so as to take the area covered by illumination, i.e., the orange rectangle in Figure 16a, as the object size to fill the overall active area of the sensor. When tiling the range images, the thin boundary of the range image with crosstalk is removed to leave the part without crosstalk, which becomes exactly the range image of the first quadrant in Figure 16a. Based on this concept, the design of illumination optics and imaging optics have to be carried out concurrently, as well as the associated post-image tiling process.

There are some other technical issues resulting from exploiting the lens array in the flash LiDAR system. For example, the alignment tolerance of both illumination optics and imaging optics will become tighter compared to when using a single-lens module. Misalignment could easily cause unexpected crosstalk or missing information. Furthermore, if the additional synchronization between the scanning illumination and the sensor went wrong, the quadrant images of a different full image will be tiled together. Distortion can be another issue in image tiling, which leads to discontinuity at the tiling boundary; however, it does not have much of an effect on depth sensing. Nevertheless, all the above-mentioned issues have corresponding solutions with existing technology and do not impose fundamental limits on the development of the proposed architecture using flash LiDAR.

## 6. Conclusions

A combination of multi-view imaging optics with scanning illumination optics has been proposed to enhance the resolution of a flash LiDAR system by only modifying the optics of currently available devices. With proper light shielding and depth calibration procedures, a tiled range image has been successfully made to prove the feasibility. The remaining crosstalk issue has also been investigated with a corresponding resolving scheme provided. The proposed optical system is equivalent to an area scanning architecture, where each scanning position covers a certain area rather than a single point, offering a degree of freedom for trading off between resolution and frame rate, which gives a further degree of freedom in the trade-off of performance indices of ToF devices such as resolution, field of view, frame rate, etc., with the two extreme cases being single-beam scanning and direct imaging.

## Figures and Tables

**Table 1:** Specifications of AD-FXTOF1-EBZ flash LiDAR device.

Item	Content
Range	Near: 250 mm to 800 mm
	Medium: 300 mm to 3000 mm
Accuracy	<2% for all ranges
Resolution	640 × 480 pixels
Illumination source	CW 940 nm VCSEL *
Receive lens	FOV * 87° × 67° including 940 nm BPF *, F/# * = 1.2
Sensor type	CMOS

* VCSEL: vertical-cavity surface-emitting laser; FOV: field of view; BPF: band-pass filter; F/#: f-number.

**Table 2:** Design specifications for lens element.

Item	Content
Material	PMMA *
Wavelength	940 nm
FOV *	30° in diagonal
Object distance	1500 mm
Object height	401.924 mm
Image height	4.5 mm
Magnification ratio	0.0112
MTF	MTF@88.9lp/mm > 5%

* PMMA: poly (methyl methacrylate); MTF: modulation transfer function.

## Data Availability

Dataset available on request from the authors.
